# Vaccines, pharmaceutical products, and bioterrorism: challenges for the U.S. Food and Drug Administration.

**DOI:** 10.3201/eid0504.990414

**Published:** 1999

**Authors:** K C Zoon

**Affiliations:** U.S. Food and Drug Administration, Rockville, Maryland 20852, USA. zoon@cber.fda.gov


### Vaccines, Pharmaceutical Products, and Bioterrorism: Challenges for the U.S. Food and Drug Administration

**Kathryn C. Zoon**
U.S. Food and Drug Administration, Rockville, Maryland, USA

In regards to bioterrorism, the goal of the U.S. Food and Drug Administration (FDA) is to foster the development of vaccines, drugs and diagnostic products, safeguards of the food supply, and other measures needed to respond to bioterrorist threats. Many products (vaccines, therapeutic drug and biological products, food, devices, and diagnostics) regulated by FDA could be affected by bioterrorism. Pathogens or pathogen products adapted for biological warfare include smallpox (variola), anthrax (*Bacillus anthracis*), plague (*Yersinia pestis*), tularemia (*Francisella tularensis*), brucellosis (*Brucella abortus, B. melitensis, B. suis, B. canis*), Q fever (*Coxiella burnettii*), botulinum toxin (produced by *Clostridium botulinum*) and staphylococcal enterotoxin B. New products are needed to diagnose, prevent, and treat these public health threats.

FDA is participating in an interagency group preparing for response in a civilian emergency. This group includes representatives of the Department of Defense; the Veterans Administration; and components of the Department of Health and Human Services (DHHS), such as the Centers for Disease Control and Prevention (CDC), National Institutes of Health (NIH), and Office of Emergency Preparedness. In addition, FDA will be proposing standards for the use of animal efficacy data in approving new products to counter chemical and biological agents. The agency is also participating in setting a broad-based federal research agenda to facilitate the government's preparedness against bioterrorism; is identifying facilities and activities suitable for the production of biological weapons; is involved in product development, review, and testing; and is ensuring that appropriate product surveillance and sponsor compliance are executed in accordance with regulations.

Address for correspondence: Kathryn C. Zoon, Center for Biologics Evaluation and Research, Food and Drug Administration, Mailstop HFM-1, 1401 Rockville Pike, Rockville, MD 20852, USA; fax: 301-827-0440; e-mail: zoon@cber.fda.gov

FDA's regulation of medical products is based on science, law, and public health considerations (Figure 1). Research conducted at FDA (in particular at the Center for Biologics Evaluation and Research) contributing to biological warfare defense and other counterbioterrorism efforts is in the following areas: design of new vaccines (e.g., pox viruses); pathogenesis and mechanism of replication of biological warfare agents; new methods and standards to expedite the review of new vaccines and immunoglobulins (e.g., mucosal protection against a pathogen); and stem cell protection and chemokine/cytokine and angiogenic agent defense mechanisms. The development framework of most biological and traditional drug products is shown in Figure 2. The principal evaluation and research and development phases before a drug is submitted to FDA for approval can take 1 to 3 years. The clinical research and development program (investigational phase), depending on the agent and clinical indication, can take 2 to 10 years. The marketing application review period generally is 2 months to 3 years (average 1 year). Once a product is approved, long-term postmarketing surveillance, inspections, and product testing are performed to ensure the quality, safety, and efficacy of the product, as

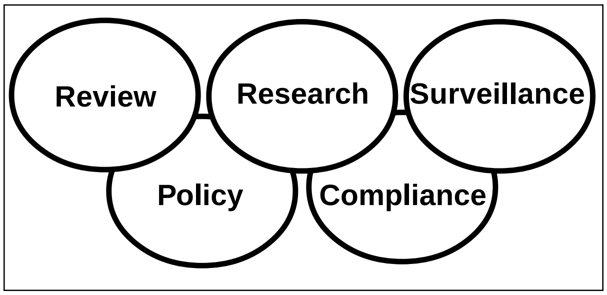

Figure 1. Regulation of medical products.

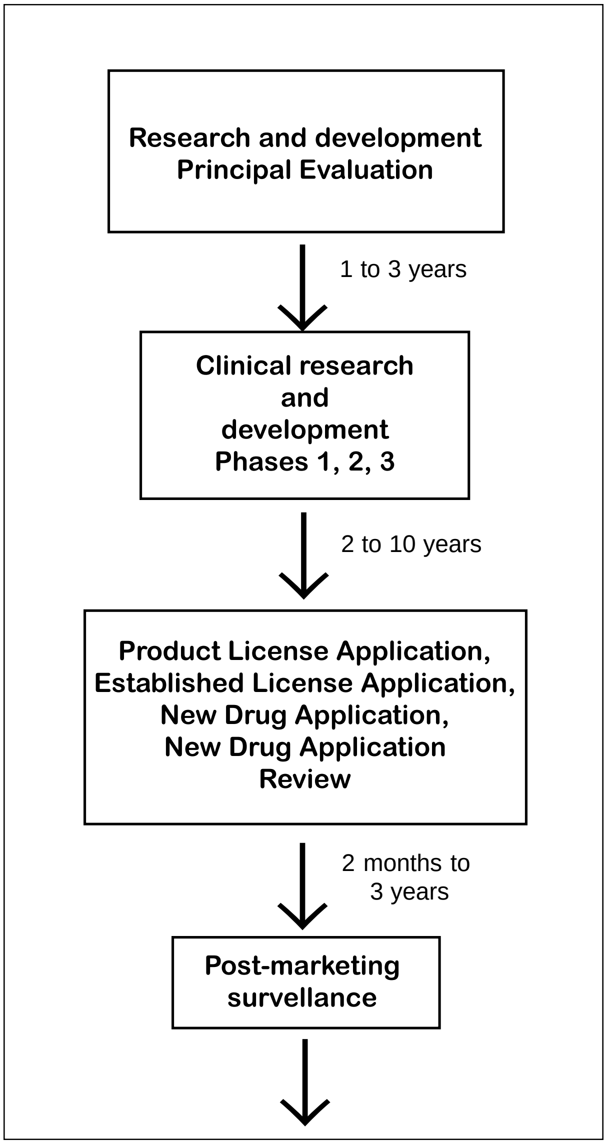

Figure 2. Development of biological and tradition drug products.

well as appropriate product labeling. Accelerating product development is important in many situations, including bioterrorism. Mechanisms for advancing medicines through the approval process have been developed for severe and life-threatening illnesses. For drugs and biologic products, these mechanisms include expedited review and fast-track development, as well as accelerated approval and priority review of marketing applications. For a priority product, complete review of marketing applications is 6 months.

Many of the biological warfare defense products pose difficult problems with regard to obtaining clinical efficacy data. For many of these infectious agents or toxins, human efficacy trials cannot be performed, as such studies would involve exposing healthy human volunteers to a lethal or permanently disabling agent without proven therapy and field trials. In most cases, such trials are not feasible because pockets of natural exposure do not exist. To address this dilemma, FDA will be proposing that the use of animal efficacy data be allowed when appropriate (1). This proposed rule would identify the types of data required. Safety, pharmacokinetic, and immunogenicity data will still be necessary in humans. Product safety will likely be evaluated in healthy human volunteers at doses and routes of administration anticipated in field use.

Some scientific considerations for animal studies include the toxic agent's pathophysiologic mechanism of toxicity and how the test drug or biologic product prevents it and the validity of the animal study endpoint in humans. In addition, data showing that drug effectiveness in animals predicts efficacy in humans would be needed. Finally, product recipients should be given follow-up after treatment to affirm product safety and efficacy.

For licensure or other approval, a biological warfare defense product must have an acceptable quality, safety, efficacy, and potency profile. Likewise, the product must have acceptable stability characteristics and be produced in compliance with current good manufacturing practices.

A case study of anthrax vaccine can serve as an example of our capability to respond to a bioterrorist threat. Only one licensed anthrax vaccine (Bioport Corp.) is available. This vaccine consists of a membrane-sterilized culture filtrate of *B. anthracis* V770-NP1-R, an avirulent, nonencapsulated strain. The culture filtrate is adsorbed to aluminum hydroxide and formulated with benzethonium chloride (preservative) and formaldehyde (stabilizer). The administration schedule consists of 0.5 ml injected subcutaneously at 0, 2, and 4 weeks, 6, 12, and 18 months, and then annually thereafter. The vaccine was licensed in 1970. The efficacy data in support of the license consisted of a single-blind, well-controlled field study (2). The vaccine efficacy was 92.5% (lower 95% confidence limit of 65%). Of the 26 cases of anthrax in this study, 21 were cutaneous and 5 (4 fatal) were inhalation (2 in the placebo group, 0 in the vaccinated group, 3 in the unvaccinated group).

In December 1985, the Federal Register (3) published the FDA's advisory panel review of the efficacy of anthrax adsorbed. The panel recommended that this product be placed in category I (safe, effective, and not misbranded) and that the appropriate license be continued because there was substantial evidence for this product.

Studies of new anthrax vaccine products are in progress. They include protective antigen–based vaccines, e.g., purified protein from *B. anthracis* culture or live-attenuated spore vaccine. Production and product testing will differ for each of these candidate vaccines. The immunogenicity of the product in humans and animal models should be assessed. The cell-mediated immunity elicited by the vaccine may also need to be evaluated. One of the immune correlates of protection of anthrax vaccines is likely to be the antibody response to protective antigen. However, the quantitative relation of antiprotective antigen antibody to protection has not been established in humans but is being investigated by the Department of Defense. Animal challenge and protection models, especially rabbit and nonhuman primate models, may be particularly useful. Passive transfer of protection, also an indication of the importance of antibodies for protection, has been observed in animal models. Therefore, human challenge protection studies and new field efficacy trials are not feasible in studying the efficacy of new anthrax vaccines. Animal challenge and protection studies against spores will be important for new vaccines based on protective antigen. Comparisons of immune responses in human cohorts receiving new or licensed vaccines should be performed.

Data should be obtained on various target populations, including adults and children, to evaluate the safety of new anthrax vaccines. Systemic and local adverse events are particularly important to monitor. For live-attenuated and vector vaccine approaches, the potential for transmission to others will be an important consideration in clinical development and use. After these vaccines are licensed and administered, the safety and adverse reactions of these vaccines should be assessed.

In conclusion, FDA will be providing a critical link in access of new medicines for biowarfare defense (Table). The expected outcomes of these activities include safe and effective products to treat or prevent toxicity of biological and chemical agents; methods to rapidly detect, identify, and decontaminate hazardous organisms; a greater ability to ensure the safety of the food supply; and a greater ability to provide appropriate medical care and a public health response.

Table. Proposed activities of the U.S. Food and Drug Administration to counter bioterrorism

1. Enhancing the expeditious development and licensure of new vaccines and biological therapeutics through research and review activities—anthrax vaccine and antisera to botulinum toxin, for example.
2. Enhancing the timeliness of application reviews of new drugs and biological products and new uses of existing products.
3. Participating in the planning and coordination of public health and medical response to a terrorist attack involving a biological or chemical agent(s).
4. Participating in the development of rapid detection and decontamination for agents of bioterrorism such as *Clostridium botulinum* toxins, *Yersinisa pestis, Bacillus anthracis*.
5. Ensuring the safety of regulated foods, drugs, medical devices, and biological products; arrange for seizure and disposal of affected products.
6. Developing techniques for detection of genetic modifications of microorganisms to make them more toxic or antibiotic- or vaccine-resistant.
7. Rapidly determining a microbe's sensitivity to drug therapy.
8. Determining the mechanism of replication and pathogenicity or virulence of identified organisms including elements that can be transferred to other organisms to circumvent detection, prevention, or treatment.
9. Enhancing adverse product reporting surveillance capabilities.

Dr. Zoon is director of the Center for Biologics Evaluation and Research (CBER) at the Food and Drug Administration. As former director of the Division of Cytokine Biology in CBER, Dr. Zoon was actively involved with regulatory issues related to cytokines, growth factors, studies on interferon purification and characterization, and interferon receptors.

### References

1. Federal Register Vol 63 #80, Monday, April 27, 1998, p. 21957.
2. Brachman PS, Gold H, Plotkin SA, Fekety FR, Werrin M, Ingraham NR. Field evaluation of a human anthrax vaccine. Am J Public Health 1962;52:632-45.
3. Federal Register. Vol 50 #240, Friday, Dec. 13, 1985, p. 51002-17.
